# Catching the engram: strategies to examine the memory trace

**DOI:** 10.1186/1756-6606-5-32

**Published:** 2012-09-21

**Authors:** Masanori Sakaguchi, Yasunori Hayashi

**Affiliations:** 1 Brain Science Institute, RIKEN, Wako, Saitama, 351-0198, Japan; 2 Saitama University Brain Science Institute, Saitama University, Saitama, 338-8570, Japan

## Abstract

Memories are stored within neuronal ensembles in the brain. Modern genetic techniques can be used to not only visualize specific neuronal ensembles that encode memories (e.g., fear, craving) but also to selectively manipulate those neurons. These techniques are now being expanded for the study of various types of memory. In this review, we will summarize the genetic methods used to visualize and manipulate neurons involved in the representation of memory engrams. The methods will help clarify how memory is encoded, stored and processed in the brain. Furthermore, these approaches may contribute to our understanding of the pathological mechanisms associated with human memory disorders and, ultimately, may aid the development of therapeutic strategies to ameliorate these diseases.

## Introduction

One of the major aims of modern memory research is to locate the physical substrate of memory (also referred to as ‘memory trace’ or ‘neural substrates of memory’) in the brain. At the beginning of the 20^th^ century, Richard Semon introduced the word, ‘engram’, to describe the memory trace, ‘Its result, namely, the enduring though primarily latent modification in the irritable substance produced by stimulus, I have called an *Engram*, ….’ [1]. Later, Karl Lashley, a pioneer in the field of behavioral neuroscience, attempted to identify the location of the engram in rodents using a maze task [2]. He systematically lesioned different parts of the brain and examined the behavioral consequences. Although he failed to locate a specific brain region where the memory trace exists, this approach has been and still is one of the most commonly used methods to understand the role of selected brain regions in memory.

Clinical studies of an epilepsy patient, known by the initials H.M., led to the accidental discovery of a milestone in memory research. To provide therapeutic relief for the epileptic seizures that H.M was experiencing, the hippocampi and amygdalae were surgically removed. Fortunately, with this treatment, H.M.’s epilepsy was cured. However, surgical intervention also produced severe anterograde and temporally-graded retrograde amnesia of autobiographical memory [3]. Subsequent studies of H.M. revolutionized our view of memory by suggesting that there are particular locations in the brain that play an essential part in memory.

The association of elementary events has been proposed to play a central role in memory [4]. In line with this notion, recent developments in memory research have focused on associative learning and memory [5]. Pavlov was well known for implementing an experimental paradigm to quantify associative memory, using a so-called classical conditioning paradigm. In classical conditioning, subjects associate two different sensory stimuli, defined as the conditioned stimulus (CS) and the unconditioned stimulus (US) [6]. The CS is a cue that is neutral but salient enough to be recognized by the subject, whereas the US is a cue that evokes an innate response in the subject, leading to an unconditioned response (UR). When the subject learns the association between the CS and the US, the subject may display a response similar to the UR upon exposure to the CS alone. This response is called a conditioned response (CR).

Many modern memory researchers have not only re-confirmed the earlier findings of Pavlov using different variants of classical conditioning paradigms, but these subsequent studies have provided many further insights into the neurobiology associated with memory (reviewed in [7]). Philips and LeDoux showed that distinct brain regions have different contributions to memory formation [8]. Using a contextual fear conditioning paradigm, they showed that the hippocampus, in cooperation with the amygdala, plays an essential role in associative learning of a specific context (CS) and a foot-shock (US). This finding is in contrast to tone-fear conditioning, where subjects learn that a neutral tone (CS) can act as a predictor of a foot-shock (US). In this paradigm the amygdala played an essential role but the hippocampus was dispensable. While this study highlighted an important functional distinction between the hippocampus and amygdala in fear memory, it was still unclear at which stage of memory (e.g., acquisition, retention, recall) each brain region was important.

**Figure 1:**
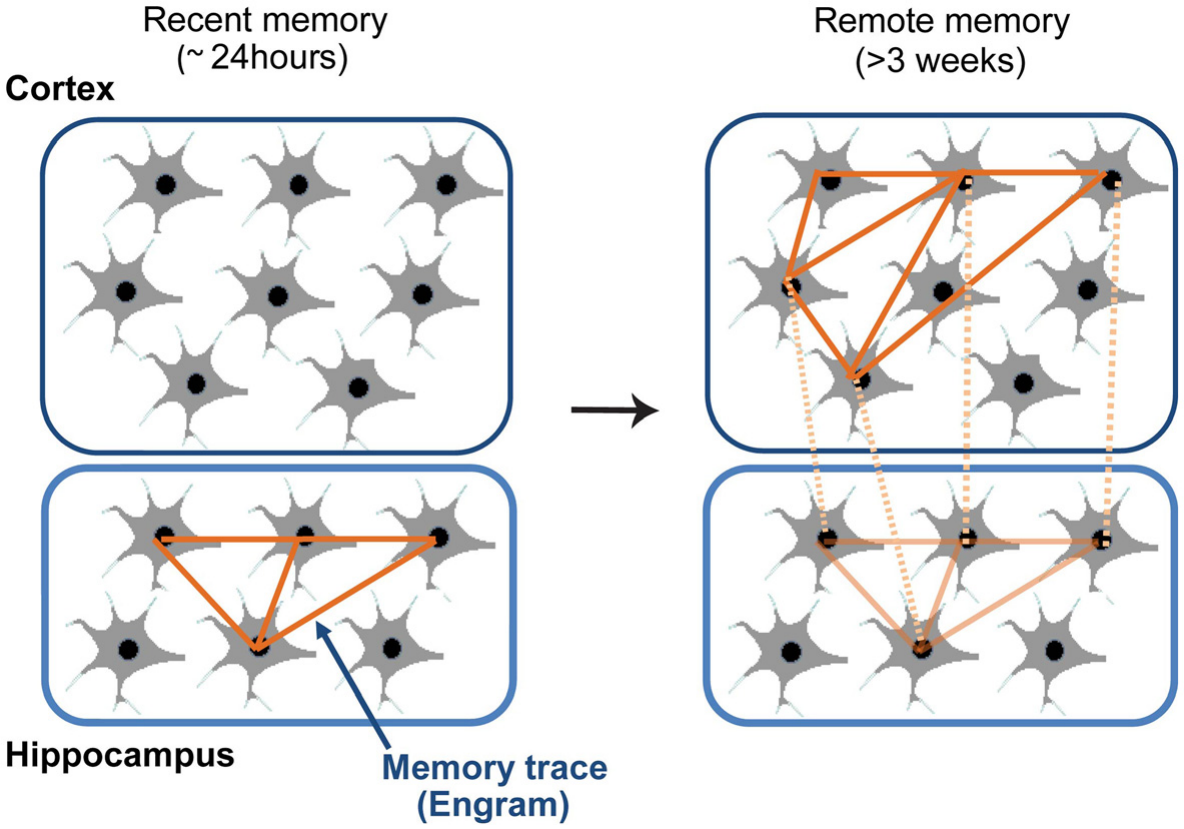
**Systems memory consolidation theory.** Autobiographical memory is initially encoded in neural ensembles in the hippocampus. After a certain period of time, neural ensembles outside of the hippocampus become responsible for the retention of memory. This dynamic system wide change is called systems memory consolidation.

Kim and Fanselow tried to examine the temporal window when the hippocampus was important for memory, by applying electrolytic lesions to the hippocampus at various time points after contextual fear conditioning [9]. Rats that were lesioned 1 day after training did not retain the contextual fear memory, whereas animals that received the lesion 28 days later retained the memory at the same level as sham operated rats. These findings were supported by numerous other studies (e.g. the case study of H.M. and other studies using pharmacological/genetic inhibition approaches or electrolytic/surgical lesions) and led to the proposal that certain types of memory are initially encoded by the hippocampus as recent memory and then gradually transferred from the hippocampus to other brain regions as remote memory [9-14]. The process of memory transfer is commonly referred to as the systems consolidation theory (Figure 1). However, whether the hippocampus is still engaged in remote memory and whether the transferred memory is the same as the original memory is still under much debate [15-21].

The obvious shortfall in the lesion studies is that surgical lesions not only destroy all locally existing structures including neuronal and glial cells but also incoming, outgoing, and even passing fibers. Also, the irreversibility of the procedure makes it difficult to determine the exact role of the lesioned brain regions at different stages of memory. Using drugs that selectively inactivate neuronal activity has some notable advantages over physical lesions and can help elucidate the relationship between biological phenomenon and memory. For example, Kandel et al. elucidated the molecular mechanisms of memory related behavior by applying drugs (e.g., cAMP or CRE oligonucleotides) onto target neurons of Aplysia [22]. Morris showed a link between long term potentiation and spatial memory by applying a pharmacological reagent that blocks LTP (AP5, an antagonist of NMDA type glutamate receptors) directly into the brains of rats [23]. Recent advances in genetic techniques (e.g. optogenetics) have enabled the manipulation of neural activity at an even higher level of sophistication and temporal control (e.g., inactivating targeted neurons in reversible manner at specific points in time [24,25]). Using these techniques, it is now possible to visualize and control specific neuronal circuits that encode associative memory [26,27].

Given these advances, in this review, we will provide an overview of recent studies that aim to allocate the memory engram at the circuit, cellular, and synaptic level and discuss the current debate, remaining questions and future perspectives.

## Visualization and manipulation of a memory trace by immediately early genes (IEGs)

### Detection of IEG products

IEGs are a class of genes that are transcribed immediately after biological events (starting in less than a minute) without requiring the expression of other genes [28,29]. Neural activity induces expression of various IEGs including proto-oncogene transcription factors such as *c-fos* and *zif/268* (also named *Egr1, NGFI-A, Krox 24*) and genes that encode synaptic structural proteins such as Arc and Homer1a. Due to their property of activity dependent transcription, immunostaining or *in situ* hybridization of IEGs allows us to identify neurons that were active during a given memory paradigm.

**Figure 2:**
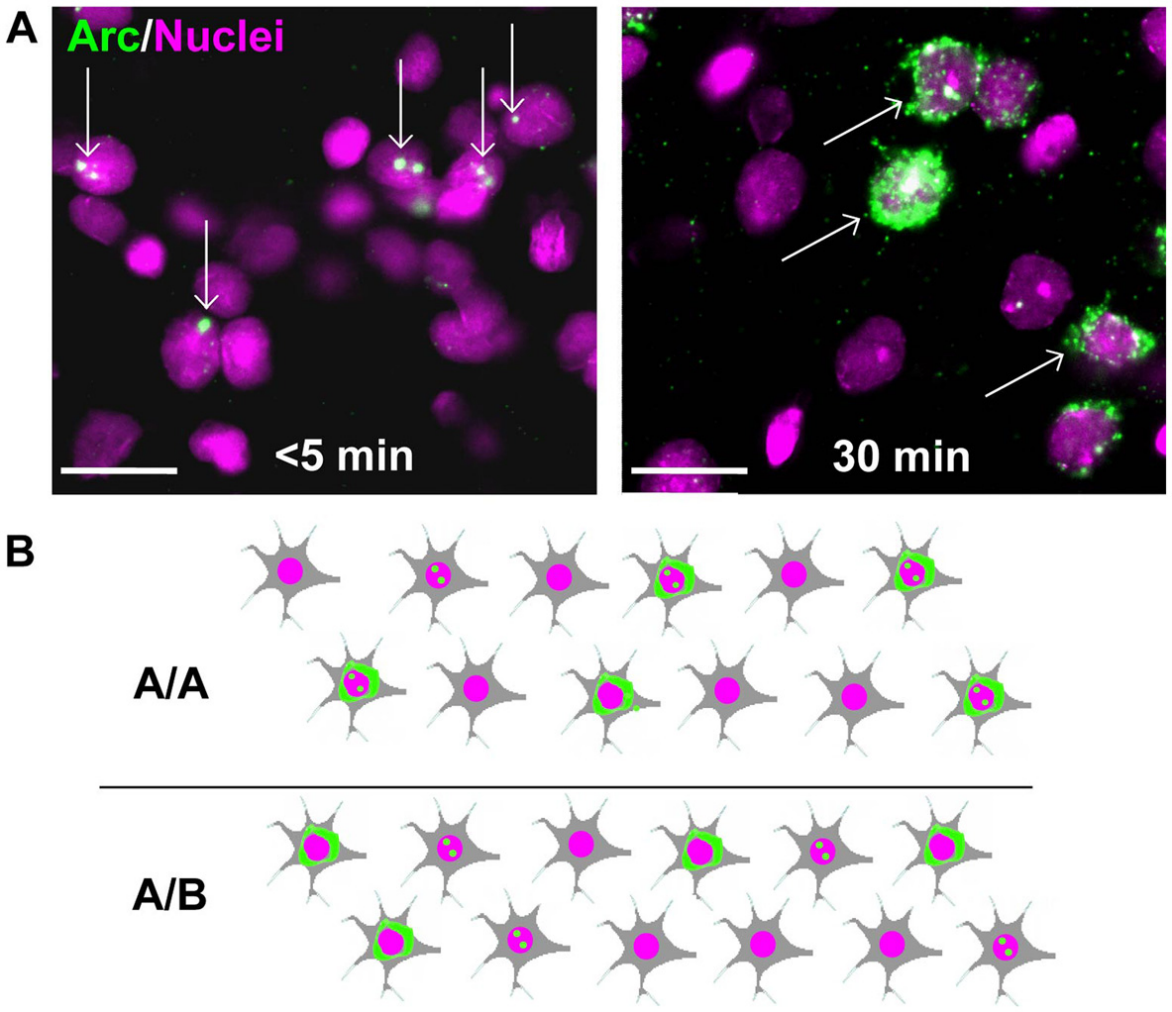
**Tagging neural ensembles by Arc catFISH. A**, After neuronal activation, Arc mRNA is initially localized in the nucleus (left panel). Within 30 minutes, the mRNA translocates outside the nucleus (right panel). **B**. The Arc expression pattern in a neuronal ensemble is depicted in two different situations. In the upper panel, the subject was exposed to context A at two time points, firstly at 30 minutes and secondly 5 minutes prior to sacrifice and fixation (A/A). In the lower panel, the animal was first exposed to context A and then to a distinct context B (A/B). Exposure to the A/A condition led to induction of Arc localization both inside and outside of the nucleus in the same neurons, while A/B exposure induces Arc in mainly two distinct populations albeit with some overlap.

The mRNA of Arc has interesting distribution dynamics, which allows one to trace activated neurons in a retrograde manner. It initially accumulates in the nuclei of neurons (<5 min) and is gradually exported to the cytosol within the next 30 min (Figure 2A). Therefore, by observing the localization of Arc mRNA, one can not only detect activated neurons but also estimate the approximate time that has elapsed after the initial activation. The method is named ‘cellular compartment analysis of temporal activity by fluorescent *in situ* hybridization’ of Arc (Arc catFISH) [30].

Using the Arc catFISH method, Guzowski et al. [28] showed that when a rat is exposed to two different environments with an interval of 30 minutes, activation of Arc in response to the exposure to each environment occurs in separate neuronal populations in the CA1 (Figure 2B, lower row). In contrast, if a rat is exposed to the same environment twice, the same neurons that were activated during the first episode were reactivated during the second episode (Figure 2B, upper row). This result suggests that the environment specific memory trace is established in the CA1. Using an associative learning paradigm, Barot et al. [31] showed that individual neurons in the basolateral nucleus of the amygdala responded to both CS and US only after the subject had learned the association between CS and US.

It is suggested that once memory is encoded, it is consolidated through off-line neuronal ensemble activity (e.g., activity during rest or sleep when there is no CS or US). Marrone et al. [32] compared neuronal ensemble activity in the hippocampus during periods of exploration and then the following rest period using the catFISH method. They found that the Arc expression pattern during the rest period partially recapitulated that of the exploration period. Hashikawa et al. [33] extended this idea by examining associative learning in the amygdala using a fear conditioning paradigm and showed that amygdala neurons that expressed Arc during conditioning also preferentially re-expressed Arc during the following rest period.

### Use of IEG promoters

**Figure 3:**
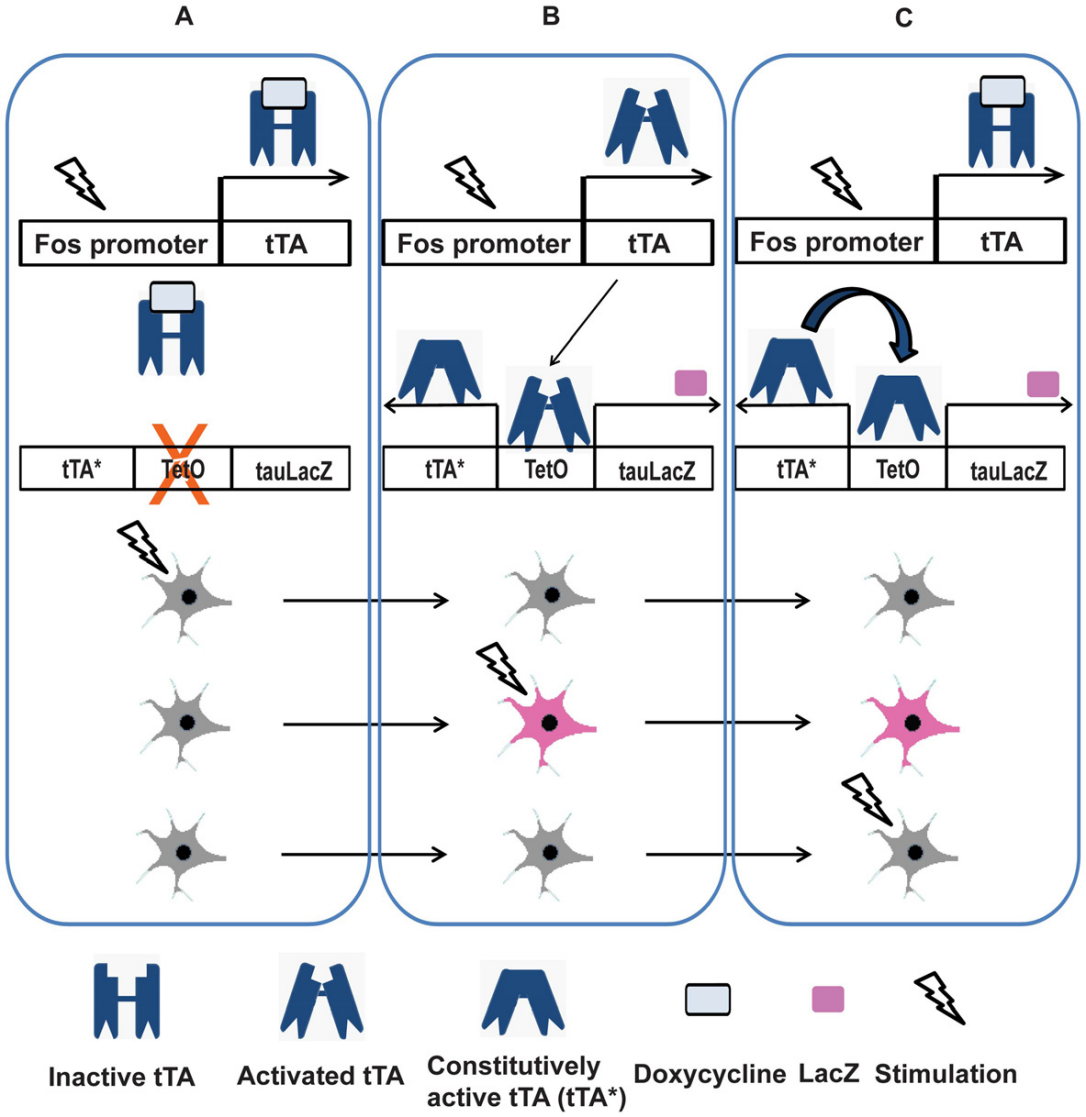
**Tagging neural ensembles using a c-fos promoter tTA/TetOLacZ-tTA mouse.** The mouse has two transgenic alleles. One allele encodes the doxycycline sensitive tetracycline transactivator (tTA) under the control of the c-fos promoter. tTA binds the tetracycline operator (TetO) sequence to induce transcription of the downstream gene, and the induction activity is suppressed by the presence of doxycycline. The other expresses the secondary transactivator (tTA*) and tauLacZ under the control of TetO. tTA* is both insensitive to doxycycline and constitutively active, therefore, once expressed, tTA* activates TetO regardless of doxycycline. **A**. tTA is expressed exclusively in neurons where the c-fos promoter is activated. However, in the presence of doxycycline, tTA activity is inhibited and hence prevents interaction with TetO. **B**. After withdrawal of doxycycline, tTA is activated and binds to TetO to induce expression of tTA* and tauLacZ protein in c-fos activated neurons (purple). **C**. After re-administration of doxycycline, tTA becomes inactivated once again but as tTA* is still present, it can maintain the expression of tauLacZ by activating TetO in the positive-feedback loop. This system thereby allows the permanent tagging of neurons activated even in the absence of doxycycline.

In addition to the observation of IEG products, the promoters for IEGs can be used to detect neurons involved in learning. Reijimers et al. [34] visualized c-fos positive neurons using a transgenic mouse line expressing **β**-galactosidase (LacZ) under the control of a c-fos promoter via a self-activating tTA-TetO system to examine the memory trace of tone fear memory in the amygdala (Figure 3). During training, LacZ was induced in some neurons in the amygdala. Interestingly, the same LacZ positive cells were also positive for Zif268 after memory retrieval, suggesting that the LacZ/Zif268 double-positive cells encoded the fear memory. A similar technique was used to induce the expression of newly synthesized AMPA type glutamate receptors (AMPAR) selectively in neurons that were activated during memory formation (i.e., c-fos activated neurons), which were then translocated to specific types of dendritic spines [35]. Recent advances in fluorescent imaging techniques allow us to examine neurons involved in memory in living subjects. A mouse line which has GFP knocked-in into the *arc* promoter locus was generated for this purpose, however, the experimental results could be confounded by a phenotype caused by the hemizygous loss of Arc gene (e.g., orientation specificity) [36]. Hence, several transgenic mouse lines with *arc* or *c-fos* promoter to drive the expression of fluorescent proteins were established that could circumvent this issue [37-41].

The induction of c-fos expression upon conditioning indicates that there is a correlation between neuronal activation and memory, but it does not necessarily prove that c-fos expressing neurons encode the memory. Koya et al. [42] provided evidence that c-fos expressing neuronal ensembles in the nucleus accumbens were involved in the memory trace that associated a specific context (CS) with cocaine administration (US). For this study, they utilized a combination of c-fos promoter-LacZ transgenic rats and Daun02, a prodrug that can be converted to Daunorubicin by LacZ. Daunorubicin inactivates neurons by reducing Ca^2+^ dependent action potentials [42-44]. Using this combinational approach, c-fos activated neurons (i.e., LacZ-positive neurons) could be selectively inactivated by Daun02 injection. In their study, learning of a context-drug administration memory induced c-fos expression in neuronal ensembles in the nucleus accumbens of rats. Subsequent administration of Daun02 reduced the context-specific cocaine-induced psychomotor sensitization, confirming that neurons that were activated during learning were also involved in recall of the memory that associated the context with cocaine administration.

Using the same c-fos-LacZ rat, Bossert et al. found neural ensembles that mediated a context-induced relapse to heroin addiction in the ventral medial prefrontal cortex [45]. These results suggest that the c-fos promoter can be used to genetically tag the neuronal ensemble involved in memory encoding.

**Figure 4:**
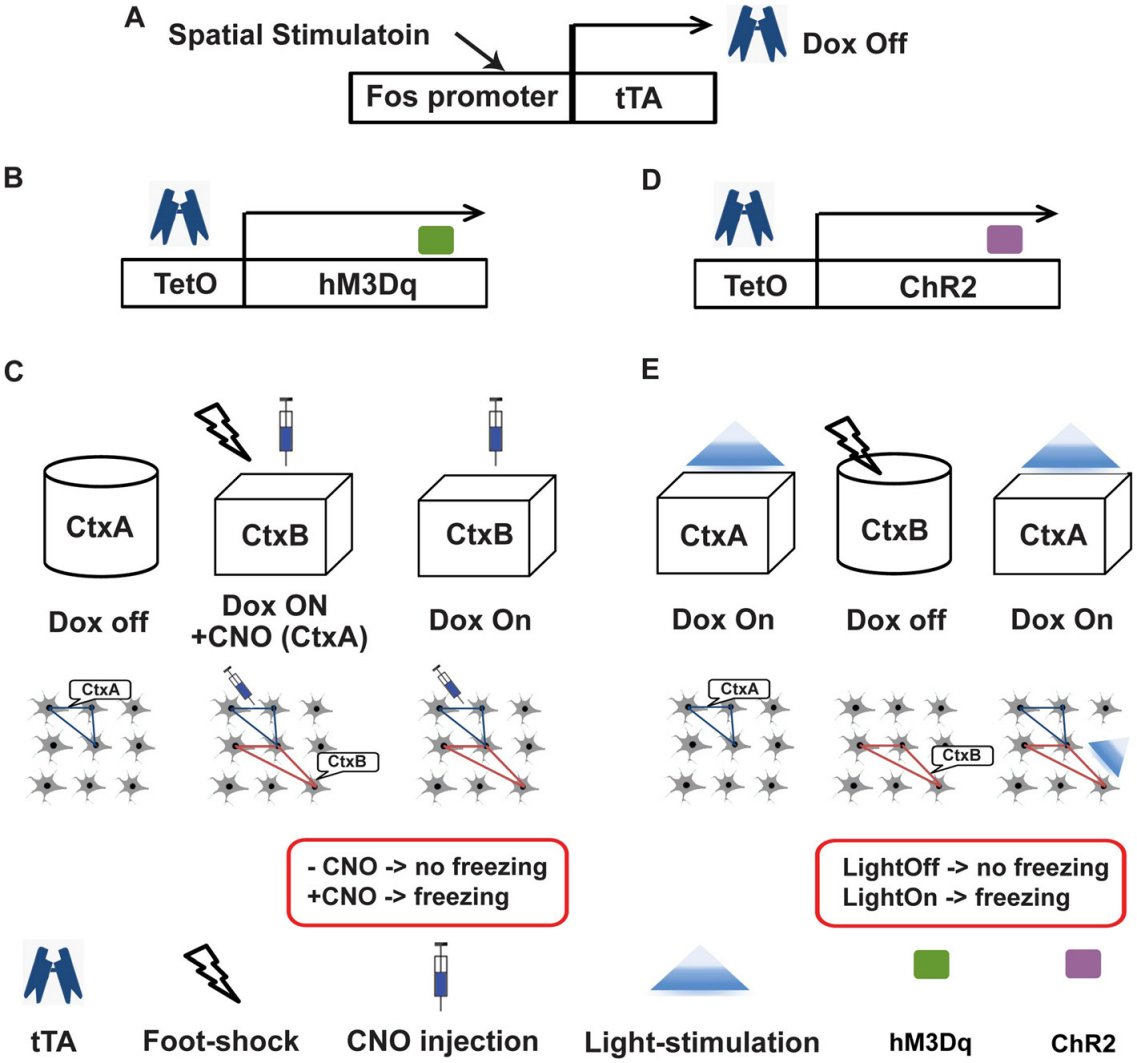
**Specific activation of neural ensembles that encode memory. A**. The two studies utilized the same c-fos promoter-tTA mouse line as shown in Figure 3. **B**. The first study used tTA to induce hM3Dq expression under the control of doxycycline. **C**. Initially, the mice were exposed to context A (CtxA) in the absence of doxycycline. The resulting neuronal hM3Dq expression is associated with cells that responded upon exposure to CtxA. Next, the mice were fear conditioned in context B (CtxB) and injected with CNO to activate neurons that express hM3Dq and recapitulate the neuronal activity pattern that was present during the earlier exposure to CtxA. In the final test session, mice were exposed to CtxB with or without CNO injection. Interestingly, only CNO injected mice were able to exhibit memory recall (by displaying freezing behavior). **D**. This study utilized a combined approach consisting of *c-fos* promoter-tTA mice and an AAV vector that carries a gene expression cassette for ChR2 under the control of TetO. **E**. First, the mice were habituated to CtxA and light stimulation was applied. The mice were under doxycycline administration at this stage. The mice did not freeze during this stage, confirming that light stimulation alone does not induce freezing. Next, doxycycline is withdrawn to promote ChR2 expression under the control of the c-fos promoter. Then, the mice were fear conditioned in CtxB. This procedure promotes ChR2 expression in neurons that were specifically activated by the contextual fear conditioning protocol in CtxB. Finally, the mice were placed back in CtxA under doxycycline administration and ChR2 expressing neuronal ensembles were activated by light stimulation. The mice displayed freezing only when light was administered (but not by exposure to CtxA alone). Importantly, mice that were not fear-conditioned also did not show freezing in the presence of light.

More recently, Garner et al. [46] reported the generation of a synthetic memory trace by genetically tagging c-fos promoter activated neurons. They used a double-transgenic mouse line which expressed tTA under control of the c-fos promoter and an evolved G protein-coupled receptor (hM_3_D_q_) under tetracycline response element (TRE). hM_3_Dq produces neuronal depolarization in response to clozapine-N-oxide (CNO) injection (Figure 4A,B) [47]. In the mice, the expression of hM_3_Dq can act as a tag of localization pattern of the neuronal activity (i.e., pattern of c-fos promoter activation) at a given time period (i.e., off-doxycycline period). First, the mice were exposed to a novel context, context A (CtxA), to induce tagging of neurons that were specifically active in the context (Figure 4C, left). Second, the mice were exposed to another novel context, CtxB, where foot shock (US) and CNO injection (to activate hM_3_D_q_ - and hence CtxA encoding neurons) was administered (Figure 4C, center), thereby CtxA + CtxB information could be associated with the foot shock. Finally, the mice showed freezing only when both CNO injection (pseudo CtxA) and exposure to CtxB occurred simultaneously (Figure 4C, right), but not when CNO injection nor exposure to CtxB was given alone. The result suggests that the mice created a hybrid ‘synthetic’ memory of CtxA and B. Using the same c-fos promoter-tTA mouse line in combination with viral delivery of TRE-channelrhodopsin, Liu et al. [48] showed that optical re-activation of the neuronal ensemble involved in memory encoding in the dentate gyrus was sufficient to retrieve a fear memory (Figure 4A, D, E). Importantly, retrieval was not induced when the neuronal ensemble had not been associated with the US (i.e., footshock), suggesting a strong causal relationship between the expression of c-fos in neurons and the association of context and shock (please see section 3 for other studies utilizing optogenetic approaches).

cAMP response elements (CRE) is a DNA sequence which can be found in the regulatory sequences of IEGs (e.g., c-fos [49], Arc [50]). In LTP, calcium and cAMP signals converge to activate cAMP response element binding protein (CREB) transcription activity by phosphorylating Serine residue 133 (CREBs133), which results in an increase in CRE mediated gene expression. Impey et al. made a transgenic mouse line, which carries tandem-repeat CRE sequences followed by a LacZ-reporter gene to monitor CRE mediated transcription activity upon LTP and memory formation [51]. Indeed, LacZ expression was well correlated with the phosphorylation of CREBs133 and induction of L-LTP in the CA1 region of the hippocampus. Furthermore, the signaling pathway that induced L-LTP enhances CRE mediated transcription. Importantly, learning of contextual fear conditioning and passive avoidance tasks increased CRE dependent gene expression in the hippocampus [52]. On the other hand, auditory fear conditioning, an amygdala dependent learning paradigm, only increased CRE dependent gene expression in the amygdala, suggesting that CRE dependent gene expression was memory type specific and that CRE up-regulation was involved not only in hippocampus-dependent but also in amygdala-dependent associative memories.

## Forced labeling and manipulation of a memory trace by CREB

The studies described above underscore the importance of the CREB transcription factor and its downstream targets in modulating the cellular response to neuronal activity that takes place during learning and memory. CREB is an interface between neuronal activity and gene transcription by converting local and transient second messenger signaling into a persistent cell-wide transcriptional modification. This feature gave rise to the idea that CREB could be used as a tool to force a neuron to encode memory.

### Labeling

**Figure 5:**
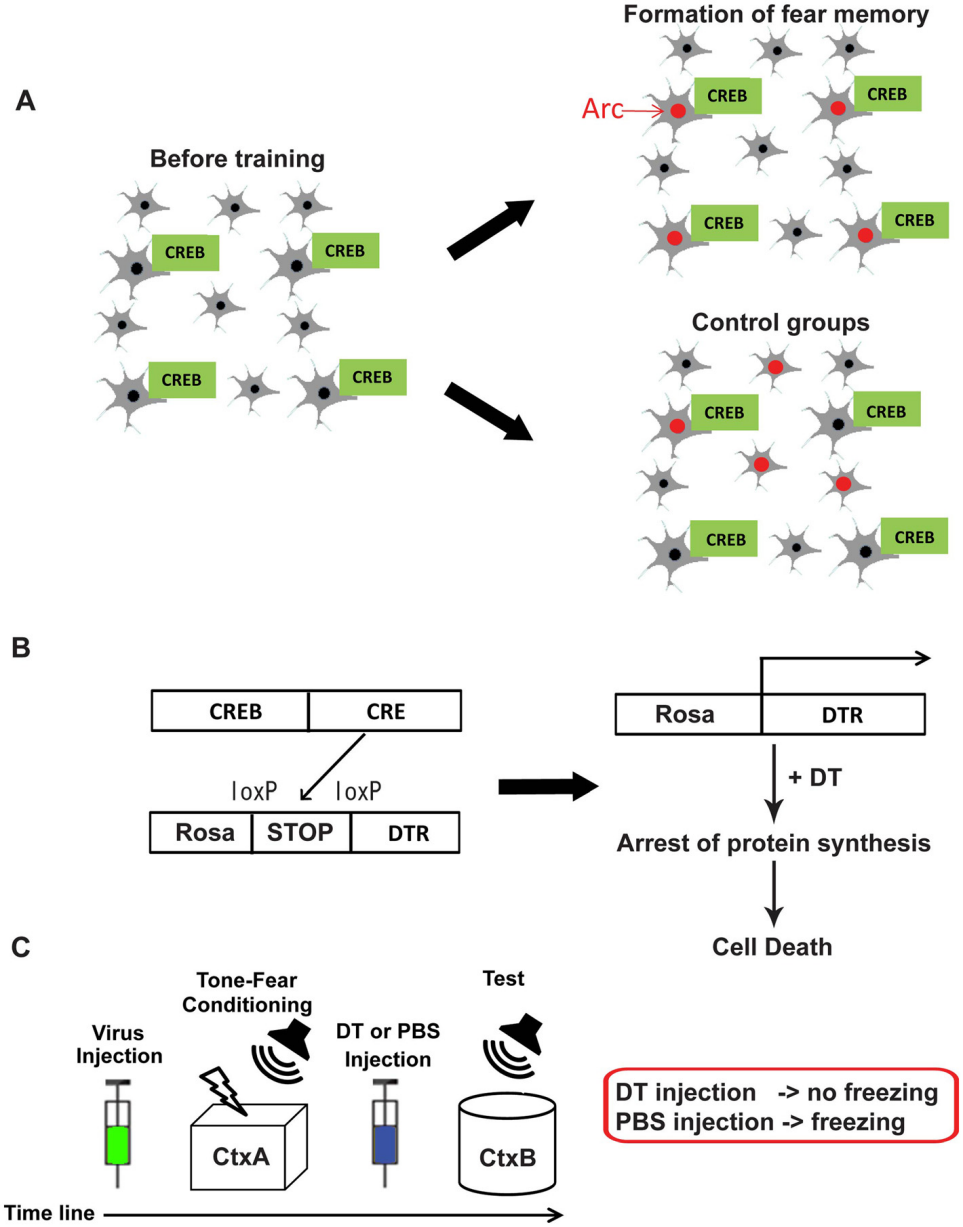
**Memory allocation by CREB. A**. CREB is overexpressed in the amygdala by a HSV vector before tone-fear conditioning (training). HSV randomly infects neurons in the amygdala. During training, the memory is allocated to neurons overexpressing CREB (CREB neurons), which results in the preferential induction of Arc expression (neuronal activity marker) in CREB neurons. Controls that did not undergo “learning” (home cage or immediate shock groups) did not show the preferential Arc expression in CREB neurons. **B**. To directly show the causal link between CREB and memory allocation, CREB neurons were selected for deletion by expression of the diphtheria toxin receptor (DTR). The system utilizes iDTR mice in which DTR expression can be induced by CRE recombinase activity. Therefore, expressing CREB-CRE using a HSV vector makes the neurons susceptible to Diphtheria Toxin (DT). **C**. Injection of DT after CREB overexpression and the subsequent fear conditioning training ablated CREB expressing neurons that resulted in a loss of the memory.

Han et al. used an auditory fear memory task to experimentally test the concept that neurons that have a relatively high CREB expression level could be recruited into a fear memory circuit [53]. Firstly, they overexpressed CREB in the amygdala, and then trained the mice in a tone-fear conditioning paradigm (tone-foot shock association memory) (Figure 5A, left). After the test, the amygdala was subjected to Arc catFISH analysis. Arc mRNA was preferentially localized to CREB overexpressing neurons rather than neighboring neurons, without changing the total number of Arc-positive cells in the amygdala (Figure 5A, right), suggesting that CREB overexpression confers the selective incorporation of CREB positive neurons in the fear memory trace by outcompeting other eligible neurons. Importantly, overexpression of CREB in home caged mice or mice that received the shock immediately after introduction to the training chamber (i.e., immediate shock group that did not form contextual memory) did not show an increase in Arc mRNA within CREB positive neurons (Figure 5A, right). This indicates that overexpression of CREB does not directly affect Arc mRNA transcription. However it should be noted that CREB has been shown to bind to a specific region of the Arc promoter, named Synaptic Activity-Responsive Element (SARE), to up-regulate Arc expression *in vitro*[50]. Epigenetic mechanisms might prevent CREB binding to SARE *in vivo* and/or a coordinated binding of other transcription factors (e.g., MEF2 and SRF), allowing *Arc* transcription only upon conjunctive presentation of both the US (footshock) and CS (tone) [54,55].

### Manipulation

This feature of CREB allows us to selectively manipulate memory encoding cells by coexpressing CREB with functional molecules. Han et al. utilized a Cre recombinase (Cre)-inducible diphtheria toxin receptor (iDTR) transgenic mouse line that allowed the selective elimination of target cells (i.e., DTR expressing neurons) [56]. They engineered a HSV vector that expresses both CREB and Cre to induce memory encoding preferentially to CREB positive neurons at the learning stage and to confer DT sensitivity to the same neurons (Figure 5B). After learning, DT was administered to selectivity ablate CREB expressing neurons. Interestingly, this process also resulted in the erasure of the newly acquired fear memory, suggesting that CREB positive neurons can selectively encode fear memory, by outcompeting other neurons in the amygdala [57].

Zhou et al. reported similar results utilizing an allatostatin receptor (AlstR)/ligand system, originally derived from insects [58]. Binding of allatostatin to heterologously expressed AlstR activates endogenous mammalian G protein-coupled inwardly rectifying K^+^ (GIRK) channels, which causes membrane hyperpolarization, thereby decreasing neuronal excitability [59]. The system allowed inducible silencing of target neurons (i.e., AlstR expressing neurons) in a reversible manner. Silencing of CREB overexpressing neurons by AlstR/ligand system resulted in a reduction in freezing during a tone-fear conditioning test, providing further evidence that CREB induces memory encoding in amygdala neurons.

The authors also examined the selectivity of memory induced by CREB. Conditioned taste aversion (CTA) memory is a type of memory known to depend on the amygdala [60]. First, mice underwent tone-fear conditioning, then later, CREB and AlstR were coexpressed in amygdala neurons, and CTA training was performed. Using this paradigm, CREB was active only during CTA training, but not during tone-fear training. The subsequent infusion of allatostatin selectively disrupted the CTA memory but not the tone-fear memory, indicating that the specific memory encoding could be induced by CREB overexpression.

What is the mechanism that enables neurons overexpressing CREB to preferentially encode memory? Electrophysiological recordings of hippocampal neurons overexpressing a constitutively active form of CREB revealed larger *N*-methyl-D-aspartate type glutamate receptor (NMDAR) currents and a greater magnitude of LTP [61]. A similar experiment performed on neurons from the nucleus accumbens indicates that CREB increases overall excitability of neurons by enhancing the Na^+^ current while suppressing the K^+^ current [62]. Morphologically, neurons overexpressing a constitutively active form of CREB have a higher density of dendritic spines [61]. Tone fear memory formation functionally strengthened thalamus-to-lateral amygdala synapses in CREB neurons but not neighboring neurons [58]. These results suggest that enhanced neuronal excitability is one of the mechanisms by which CREB mediates the induction of memory encoding in amygdala dependent memories.

In the hippocampus, overexpression of CREB rescued a spatial memory deficit in a mouse model of Alzheimer’s disease [63] and also enhanced fear memory in CFC [64]. It will be interesting to examine whether CREB overexpression can also induce memory encoding in the hippocampus or other brain regions [26].

## Optogenetics

Another powerful tool that has recently emerged in the field of memory research is the use of light-activated proteins to control neuronal activity. Boyden et al. [65] and Ishizuka et al. [66] were first to report the usefulness of channelrhodopsin, a blue light activated non-selective cation channel from green algae *Chlamydomonas reinhardtii* in enhancing spike generation. Further screening of this class of micro-organisms yielded halorhodopsin, a Cl^-^ channel and Archaerhodopsin, a proton pump, which cause neuronal hyperpolarisation upon illumination with yellow or green light, respectively. Such light activated proteins make it possible to precisely control the temporal and spatial activity of neurons in vivo [25,65,67-71].

Johansen et al. [72] showed that optogenetic stimulation of pyramidal neurons in the lateral amygdala can replace the US in a tone fear conditioning paradigm. Choi et al. [73] succeeded in inducing neuronal ensemble activity in the piriform context to control memory related behaviors using optogenetics. They showed that the same neural ensembles could be trained to evoke both appetitive and aversive behavior interchangeably.

Goshen et al. [25] examined whether the hippocampus is still engaged in the remote memory using optogenetic approaches. In contrast to previous results, where inhibition of hippocampal activity at remote time points resulted in no apparent effect in fear memory retrieval [9,12,13], inhibition of the activity of CA1 αCaMKII-positive neurons using eNpHR3.1, an improved version of halorhodopsin, specifically during the memory retrieval test resulted in a reduction in freezing not only at recent but also at remote time points. Interestingly, inhibition of activity 30 minutes before the test abolished the above effect (i.e., reduction in freezing), which is in agreement with other reports that utilize the other methods (e.g., physical, pharmacological, and genetic lesions) [9,12,13]. These results showed that the hippocampus is still engaged after memory consolidation, and highlight the higher temporal resolution of optogenetic approaches over other more conventional approaches.

## Catching the memory engram at the level of the synapse

Hebb outlined a computational model proposing that memories may be encoded by the associative activity of connected neurons at synapses, the synaptic plasticity. The proposal was substantiated by the discovery of LTP; a prolonged strengthening of the efficiency at synapses. Although its molecular mechanisms are not fully understood and may differ amongst different regions of the brain, LTP is generally considered to involve two key phenomena. One is to increase the number of AMPARs, leading to an increase in the efficiency of transmission [74,75]. For example, Rumpel et al. showed that blocking LTP by preventing synaptic trafficking of GluR1 AMPARs in neurons of the lateral amygdala can led to an impairment in memory encoding of cued fear conditioning in rats [76]. The other is to increase the size of dendritic spines, where synapses reside [77,78].

**Figure 6:**
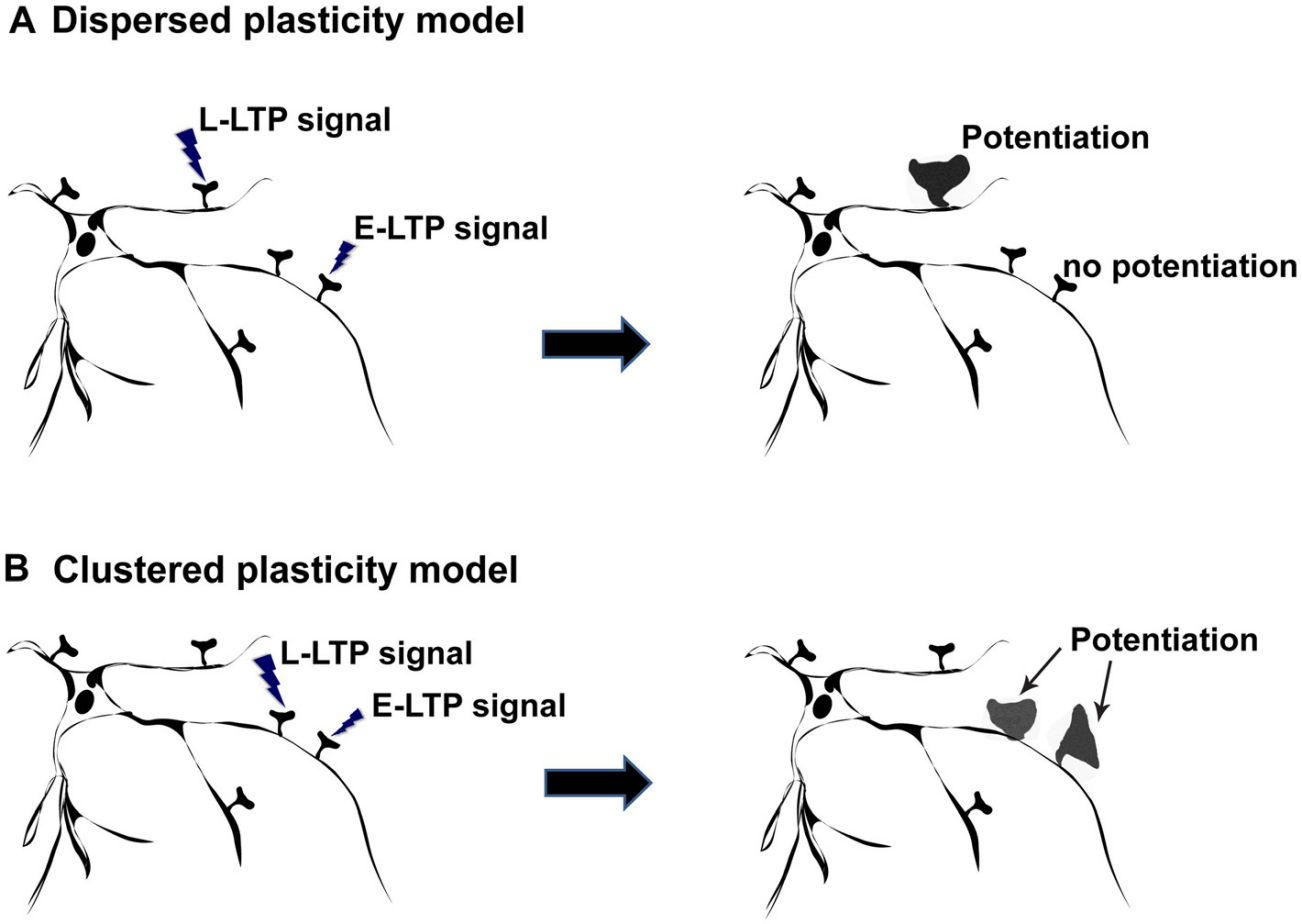
**Two models of synaptic plasticity.** Synaptic stimulation that induces long-term LTP (L-LTP stimulation) promotes the expression of Plasticity Related Proteins (PRPs), but stimulation that induces only early-phase LTP (E-LTP) does not. A recent hypothesis has suggested that activated synapses become labeled or ‘tagged’ by certain molecules (though the identity of these molecules is currently unclear). It has been proposed that once PRPs reach an activated synapse, the molecular ‘tags’ can capture the PRPs and in turn mediate the induction of L-LTP even when the synapse only receives E-LTP stimulation. This is called the Synaptic Tagging and Capture theory. Now the theory has generated two models to take into account the spatial localization of participating synapses. **A**. Dispersed plasticity model. In the model, there is no significant bias in the distribution of activated synapses among the dendritic branches of a single neuron within the PRPs expression time window. Therefore, most of the time PRPs may not be able to reach other potentiated synapses within the time window. **B**. Clustered plasticity model. If the activated synapses tend to be in close proximity to each other within the time window, PRPs can translocate to other potentiated synapses to induce L-LTP. The model also predicts a non-linear increase in the probability of neuronal firing by sharing molecules that facilitate the firing among clustered synapses (not shown here). Note that for illustrative purposes, L-LTP is depicted as an increase in the size of a synapse.

The distribution of potentiated synapses, synaptic engram, on a neuron has been largely unknown. Govindarajan et al. [79] proposed two possible patterns of distribution, a clustered plasticity model, in which the synaptic engrams of given learning paradigm are clustered in a close proximity on a dendrite and a dispersed plasticity model where the synaptic engrams are randomly distributed within the dendritic arborization (Figure 6).

The synaptic engram can be visualized by detecting the underlying molecular mechanisms of synaptic plasticity. Ca^2+^ imaging offers a functional readout of synaptic responses in near real time. It revealed that coincidental synaptic input occurs on the scale of around 10 μm on a single dendrite [80,81]. A study using a pH-sensitive GFP-tagged AMPA receptor showed that the synapses, in which AMPA receptors were newly inserted, formed clusters through a NMDA-R dependent mechanism, adding further support to the clustered plasticity model [80]. Förster resonance energy transfer (FRET) is a sensitive method to determine if two fluorophores are within a small distance of each other. Sensor molecules, such as CaMKII (a major player in LTP) [82-85], and actin (a major synaptic structural protein) [78,86], were engineered to visualize the synaptic engram using FRET.

The next question to address will be whether the clustering is related to the encoding of information that could impact animal’s behavior (e.g., CS and US), and ultimately, whether the clustering is necessary and essential for associative memory. Using transcranial two-photon microscopy, Fu et al. showed a correlation between the extent of motor learning and the clustering of new spines in the motor cortex. Motor learning facilitated clustered spine formation, whereas new spines tend to avoid regions with existing spines in control conditions [87]. Lai et al. [88] examined the correlation between the amount of synapse turnover and behavioral changes during a fear conditioning paradigm. Fear learning eliminated spines in cortical neurons whereas fear extinction induced the formation of new spines on the same branch where the spine elimination took place. Importantly, re-conditioning after extinction resulted in the selective elimination of synapses that were newly formed during extinction, suggesting that the newly formed synapses could represent the process of extinction. It will be interesting to know whether such synaptic changes are necessary and/or sufficient to change fear memory [88].

If the synaptic engram is clustered, what is the underlying mechanism? Using the size of dendritic spines as an index for transmission efficiency, Harvey et al. demonstrated in hippocampal slices that a spine that received subthreshold stimulation which normally induces only a transient enlargement can be enlarged persistently by combining it with suprathreshold stimulation of a nearby spine [70]. Moreover, the amplitude of excitatory postsynaptic potential (epsp) supralinearly sums up when stimuli are given to adjacent synapses [89]. Importantly, the efficiency of the cross-talk between the synapses is governed by the distance between two synapses and the time interval between stimuli [70,90].

These results suggest that there is signaling cross-talk between nearby dendritic spines. The imaging of movement of synaptic proteins using a photoactivatable GFP revealed that synaptic proteins are indeed shared between neighboring synapses [91,92]. FRET imaging revealed that the activity of *ras* induced in a single spine by glutamate uncaging can spread to neighboring spines [93]. These observations suggest that molecules activated at one synapse can spread to nearby synapses and such sharing may underlie the mechanisms of the cross-talk of synaptic plasticity between nearby synapses.

### Future directions

Researchers hope to clarify the mechanisms of learning and memory, and ultimately to apply the techniques and knowledge to treat memory related disorders in humans [94,95]. Recent findings obtained from studies examining the mechanisms of reconsolidation in rodents could potentially be transferred to aid clinical applications in humans to attenuate/prevent the return of learnt fear [95,96]. Such studies highlight the importance of understanding the basic mechanisms of memory to aid the establishment of viable strategies that can provide therapeutic relief to sufferers of memory disorders [95-97]. To clarify the mechanisms of learning and memory, we have to identify neuronal ensembles that encode the memory and to selectively manipulate them and observe its behavioral outcome. The main advantage of the methods discussed in this review is that they are able to selectively target memory-encoding neurons, whereas other conventional methods (such as pharmacological or surgical lesions, transcranial magnetic stimulation) cannot. At the same time certain technological advances need to be made to enable the efficient and safe delivery of genes to the human brain. The recent revival of virus based gene delivery methods [98,99] and the establishment of a method to access the deeper regions of the intact human brain [100] could provide a foundation for the future development of therapeutic strategies for the treatment of human memory disorders by directly and selectively manipulating memory encoding neurons.

## Competing interests

The authors declare that they have no competing interests.

## Authors’ contributions

MS and YH prepared the manuscript. Both authors read and approved the final manuscript.
